# Electrophysiological evidence for adult age-related sparing and decrements in emotion perception and attention

**DOI:** 10.3389/fnint.2012.00060

**Published:** 2012-08-23

**Authors:** Joshua W. Pollock, Nadia Khoja, Kevin P. Kaut, Mei-Ching Lien, Philip A. Allen

**Affiliations:** ^1^Department of Psychology, University of AkronAkron, OH, USA; ^2^Department of Psychology, Oregon State UniversityCorvallis, OR, USA

**Keywords:** aging, emotion perception, automaticity, ERPs, psychological refractory period

## Abstract

The present study examined adult age differences in processing emotional faces using a psychological refractory period paradigm. We used both behavioral and event-related potential (P1 component) measures. Task 1 was tone discrimination (fuzzy vs. pure tones) and Task 2 was emotional facial discrimination (“happy” vs. “angry” faces). The stimulus onset asynchrony (SOA) between the two tasks was 100, 300, and 900 ms. Earlier research observed larger age deficits in emotional facial discrimination for negative (angry) than for positive (happy) faces (Baena et al., 2010). Thus, we predicted that older adults would show decreased attentional efficiency in carrying out dual-task processing on the P1 (a component linked to amygdalar modulation of visual perception; Rotshtein et al., 2010). Both younger and older groups showed significantly higher P1 amplitudes at 100- and 300-ms SOAs than at the 900-ms SOA, and this suggests that both age groups could process Task 2 faces without central attention. Also, younger adults showed significantly higher P1 activations for angry than for happy faces, but older adults showed no difference. These results are consistent with the idea that younger adults exhibited amygdalar modulation of visual perception, but that older adults did not.

## Electrophysiological evidence for adult age-related sparing and decrements in emotion perception and attention

Some studies with younger adults have shown that early emotion perception of angry faces does not require attentional resources, suggesting that some emotion perception is automatic (e.g., Shaw et al., 2011). However, there is evidence suggesting that for some older adults, emotional processing of negative stimuli (Leigland et al., 2004; Baena et al., 2010) and positive stimuli (Allen et al., 2011) is compromised (relative to younger adults). We hypothesize that this is the result of age-related changes in the ventral affective system (see Dolcos et al., 2011). The ventral affective system is hypothesized to be a reflexive system involving early emotional evaluation in threat perception (including the visual cortex, fusiform gyrus, amygdala, and ventromedial prefrontal cortex [VMPFC]), whereas the dorsal attentional stream is thought to involve later “cognitive” executive functions (the frontoparietal attentional system) (Dolcos et al., 2011; see also, Corbetta et al., 2008). Evidence consistent with a ventral affective system deficit is that older adults show deficits in: discriminating emotional faces—especially negatively valenced emotional faces (Baena et al., 2010), emotional decision making (Denburg et al., 2005), and emotionally linked episodic memory (Allen et al., 2005, 2011). These are known symptoms of individuals with VMPFC damage (Bechara et al., 2000; Denburg et al., 2005) and/or amygdalar deficits (Leigland et al., 2004).

The goal of the present study is therefore to examine potential age differences in emotional processing. Similar to Shaw et al. (2011), we used Psychological Refractory Period (PRP) paradigm and measured the event-related potential (ERP) elicited by emotion stimuli. However, while Shaw et al. examined spatial attention automaticity using the N2pc ERP component, we examined attentional automaticity across age (younger and older adults) using the P1 ERP component. The P1 component (measured at the O1 and O2 electrode sites) is a visual perceptual response known to be modulated by the amygdalar function with emotional faces (Rotshtein et al., 2010). The P1 component is a particularly sensitive measure of emotional processing because Rotshtein et al. isolated the P1 ERP effect on epilepsy patients. They compared healthy controls, individuals with medial temporal lobe epilepsy (MTLE) surgery that spared the amygdala (MTLE-control), and individuals with MTLE surgery that resulted in amygdalar damage (MTLE-amygdala). The MTLE-amygdala patients (with damage to the amygdala) showed no appreciable P1 effects to emotional faces, but the MTLE-control and healthy participants did show large P1 effects to emotional stimuli (e.g., fearful vs. neutral faces). It is important that the larger P1 effect for fearful faces than for neutral faces in the two control groups was not the result of general perceptual activation because inverted faces showed no increased positivity for the fearful faces for the P1 component. Also, Holmes et al. (2008, 2009b) used the P1 component to study emotional facial discrimination in individuals with low and high trait anxiety. Consequently, there is evidence that the P1 ERP is a measure of perceptual processing and is generated by extrastriate visual cortex and fusiform gyrus (Di Russo et al., 2002; Amaral, 2003; Phelps and LeDoux, 2005). Critical to the present study is the finding that the amygdala appears to modulate perceptual processing when a visual stimulus has an emotional valence (Holmes et al., 2008, 2009a,b; Rotshtein et al., 2010). An additional question to be addressed in the present study is whether increased adult age modulates exogenous attention involving emotional stimuli [what Dolcos et al. (2011) termed the ventral affective system] in a reflexive manner. Our present working hypothesis is that if older adults exhibit a deficit in ventral affective processing, then they should show a reduced P1 emotional valance effect relative to younger adults. We also predict that this age difference should be particularly salient when central attentional resources are engaged by another non-emotional task (in the present study, Task 1).

### Psychological refractory period paradigm

We aimed to determine if emotional faces (Task 2) can still be processed even when central attentional resources are engaged by the processing of another task (Task 1) and how the processing is modulated by age. We used the PRP paradigm (Telford, 1931; Welford, 1952; Pashler, 1984), which is a widely used method for the examination of dual-task processing. In this paradigm participants are required to perform two tasks (Task 1 and Task 2) for which the stimuli are separated by a variable time interval, which is known as the stimulus onset asynchrony (SOA). The common finding is that Task 2 performance tends to decline as SOA decreases—and this phenomenon is known as the PRP effect (Telford, 1931; Pashler, 1984).

**Figure 1:**
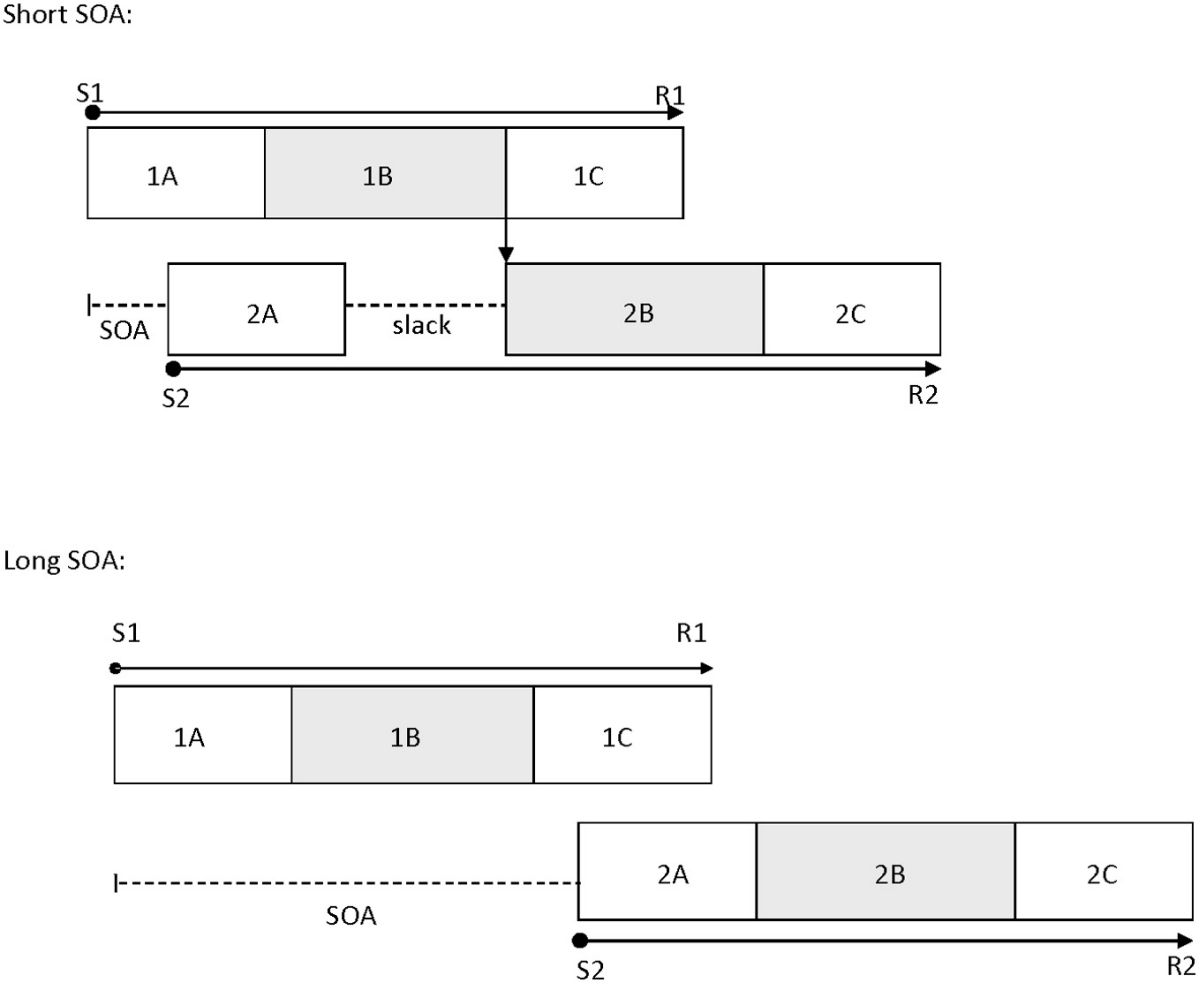
**The temporal relations between processing stages of Task 1 and Task 2 at a short SOA (top panel) and a long SOA (bottom panel) in the psychological refractory period paradigm, as suggested by the central bottleneck model.** This model assumes that perceptual and response initiation/execution stages of Task 2 can operate in parallel with any stage of Task 1, but that central stages of Task 2 cannot start until central stages of Task 1 have been completed. 1A, 1B, and 1C are the perceptual, central, and response initiation/execution stages of Task 1, respectively. 2A, 2B, and 2C are the corresponding stages for Task 2. S1: stimulus for Task 1; S2: stimulus for Task 2; R1: response for Task 1; R2: response for Task 2; SOA: stimulus onset asynchrony.

Pashler (1984) proposed the central bottleneck model to account for the PRP effect (see also Welford, 1952). The model postulates that central processing stages, such as response selection, for Task 1 and Task 2 do not operate in parallel and are instead processed serially. However, peripheral processing stages, such as perceptual encoding, can occur in parallel with all the other stages. The variable of SOA is theorized to measure the duration of the central attentional bottleneck (see Figure 1). This is because the basic assumption of this model is that Task 1 response selection must be completed before Task 2 response selection can begin. At long SOAs, there is enough time to complete Task 1 response selection before Task 2 response selection begins, so there is no bottleneck. However, at short SOAs, Task 2 is presented before Task 1 response selection is complete, and this results in a delay before Task 2 response selection can begin. On the other hand, Task 1 performance typically is unaffected by SOA because response selection for this task is completed before that of Task 2, so there is no delay (Pashler, 1984; Lien and Proctor, 2002; Ruthruff et al., 2009).

The locus-of-slack logic is a common method used to determine which operations are subject to this central bottleneck (Schweickert, 1978). According to this logic, if the manipulated Task 2 variable affects the stages prior to the bottleneck, then the effects of the Task 2 variable should be much smaller at short SOAs than at long SOAs, reflecting in an underadditive interaction between its effect and SOA (i.e., the slack effect). However, if the manipulated Task 2 variable affects the stages during or after the bottleneck, then the effects of the Task 2 variable will be additive with the SOA effect. A number of processes are indicated to be subject to the bottleneck, including response selection (Lien et al., 2002), word identification (Lien et al., 2008), memory encoding (Jolicoeur, 1998), mental rotation (Ruthruff et al., 1995), memory retrieval (Carrier and Pashler, 1995), and difficult perceptual judgments such as box-width judgment (Johnston and McCann, 2006). Due to the relatively wide variety of processes which are influenced by the bottleneck, the overall general resource attributed to all of them is commonly referred to as central attention (Johnston et al., 1995). However, some highly skilled tasks like word frequency effects in visual word recognition have been shown to exhibit slack effects (Allen et al., 2002; Lien et al., 2006).

Another (perhaps more direct) method of measuring parallel processing in the PRP paradigm was to use the ERP measure, which was applied by Shaw et al. (2011). Shaw et al. used a dual-task paradigm in which Task 1 involved two-choice tone discrimination (pure vs. fuzzy tones). For Task 2, one happy face and one angry face were presented adjacent to each other. This study had some similarities to the study of Tomasik et al. (2009) that used a behavioral version of this PRP paradigm and failed to observe slack effects for difficulty effects in emotional facial discrimination. That is, Tomasik et al. found additivity between emotional facial discrimination difficulty effects and SOA, suggesting that emotion perception was not automatic. However, the Shaw et al. study did not directly test for emotional discrimination and difficulty effects. Instead, their participants were asked to decide the gender (Experiment 1) or spatial location (Experiment 2) of a given facial emotion (emotion type was a between-subjects variable and facial emotion was easy to determine in Shaw et al.). The rationale for the Shaw et al. study was that behavioral measures of performance such as reaction time (RT) and accuracy might not be sensitive to early processing, but that electrophysiological-based ERP measures might be more sensitive to this early type of processing.

Shaw et al. (2011) used SOAs between Task 1 and Task 2 of 50, 200, and 1000 ms. They reasoned that if the shift of spatial attention to the targeted facial emotion (as indexed by the N2pc effect in ERPs) could occur without central attention resources, then N2pc effects linked to this face should not appreciably differ across SOA. That is, N2pc effects should be approximately constant across SOA. Alternatively, if this shift in spatial attention demands central attentional resources, then the N2pc effect should be delayed or attenuated at short SOAs (in an analogous manner to latencies from Task 2 being prolonged by 200–400 ms in the PRP effect). In contrast to this prediction, Shaw et al. observed statistically equivalent N2pc amplitudes at all three SOAs, with the effect elicited by angry faces being more pronounced than the effect elicited by happy faces, suggesting that emotion perception can be processed automatically (i.e., without central attention) for younger adults. Even though there was not an Emotion Type × SOA interaction in Shaw et al.'s study (suggesting a similar pattern for N2pc effects across SOA for both angry and happy faces), the N2pc amplitudes across all three SOAs were greater for angry faces than for happy faces—suggesting the attentional bias toward angry faces (also see Holmes et al., 2009a; but see Brosch et al., 2011). Different from Shaw et al. (2011), we examined directly emotion perception by asking participants to determine whether a single human face was “happy” or “angry”. Also, we used the P1 component (Rotshtein et al., 2010) instead of the N2pc (Luck and Hillyard, 1994) component used by Shaw et al. to assess emotional perception during dual-task processing because the P1 component is thought to measure amygdalar modulation of visual perceptual responses to human faces that differ in emotional valance (Holmes et al., 2008, 2009a,b; Rotshtein et al., 2010) (in our case, the emotional valence effect). However, the same logic holds for the P1 component as the N2pc component—except that we were interested in whether participants can begin Task 2 response selection of a single emotional face before completing Task 1 (pure vs. fuzzy tone) response selection. Based upon Shaw et al. (who observed a much stronger N2pc effect for angry faces than for happy faces—see their Figures 2 and 3) we predict an attentional bias for angry faces, at least for younger adults.

### Emotional processing in the brain

Adaptive behavior tends to rely on fast recognition of cues from the environment to establish threat or safety, and one such cue is facial expression (Fitzgerald et al., 2006). Research has suggested that humans are particularly efficient at processing human emotional expressions (Vuilleumier, 2000, 2002; Frischen et al., 2008). Vuilleumier et al. (2001) and Anderson et al. (2003) both observed amygdalar responses to facial expressions that seemed to be independent of attentional modulation using fMRI methods. Also, as noted above, Shaw et al. (2011) found very early N2pc activation using ERP methods for emotional faces that almost certainly was a reflexive effect, and this suggests that these early emotional effects were modulated by the amygdala. Overall, then, these results provide strong evidence that the processing of facial emotional processing can have early effects on attention. However, in the following section we will develop a more thorough model that can account for apparent early reflexive, affective processes, and later cognitive processing involved in selective attention.

As noted earlier, there is accumulating evidence for an early, reflexive ventral affective system that is modulated by affective valences and a later, controlled-process dorsal attentional stream that is cognitive in nature (Corbetta et al., 2008; Dolcos et al., 2011). The ventral affective system includes the amygdala and VMPFC and is likely a “survival” system (Allen et al., 2008). This system is likely what is referred to as exogenous attention for emotional stimuli. This system monitors incoming sensory information for potential threat and can “disengage” existing cognitive attention toward an incoming perceptual threat if such a threat is encountered (and the same process could occur if incoming information with a positive emotional valence suggested available safety). For example, if an individual steps out onto a crosswalk (directed by cognitive attention) and then automatically jumps back onto the sidewalk because exogenous attention has detected a rapidly approaching car that has run a red light, this would be an example of the ventral affective system (exogenous attention) “grabbing” attention away from endogenous cognitive attention. The dorsal attentional stream involves “endogenous” attention and is thought to be mediated by the dorsolateral prefrontal cortex, the anterior cingulate cortex, and the frontoparietal attentional pathway (Corbetta et al., 2008; Dolcos et al., 2011).

The PRP paradigm allowed us to “peek into the black box” of attentional dynamics between these two systems. The most parsimonious interpretation of Shaw et al. (2011) results in which short SOAs resulted in the same amplitude N2pc effect as longer SOAs is that the ventral affective system was able to process Task 2 negative emotions in parallel with Task 1 response selection. In the present study, we predict that the same pattern of results in P1 effects will occur for younger adults, but that older adults will show attenuated P1 emotional valence effects due to a deficit in the ventral affective system. The rationale for this hypothesis will be further developed in the next section.

### An emotion perception deficit model of aging

There have been many aging studies that examined behavioral and neuropsychological assessment tasks associated with ventral affective system function (Lamar and Resnick, 2004; Allen et al., 2005, 2011; Denburg et al., 2005; Baena et al., 2010). Also, Lamar et al. (2004) found fMRI evidence of an orbitofrontal cortex deficit for older adults (using a delayed match and nonmatch to sample paradigm), Fjell et al. (2009) found a drop in longitudinal MRI volume in healthy aging for the amygdala, and especially critical to the present study, St. Jacques et al. (2010) found impaired functional connectivity with the amygdala and visual cortical areas in older adults (relative to younger adults). Thus, past research has suggested multiple possibilities as to why older adults have less efficient emotional processing than younger adults. Some research has suggested that older adults may exhibit neural degeneration of the amygdala relative to younger adults (Leigland et al., 2004; Fjell et al., 2009) and that this results in different areas of the brain, such as the VMPFC, compensating for this loss. However, studies have also reported age-related decline in tasks associated with VMPFC function (Allen et al., 2005, 2011; Denburg et al., 2005; Baena et al., 2010; although see MacPherson et al., 2002). Indeed, Timpe et al. (2011) found that older adults with emotional decision-making deficits showed a reduction in white-matter intactness in the frontal cortex (relative to older adults without emotional decision-making deficits) as measured by diffusion tensor imaging. Thus, there are age-related deficits for either amygdalar processing or VMPFC processing, or both based on results from imaging and behavioral studies (e.g., Lamar et al., 2004; Allen et al., 2005, 2011; Denburg et al., 2005; Fjell et al., 2009; Baena et al., 2010; St. Jacques et al., 2010; Timpe et al., 2011). We hypothesize that these imaging and behavioral results are consistent with an *emotion perception deficit model* of aging. Specifically, it is proposed that older adults exhibit either structural of functional deficits that make it more difficult for the amygdala to modulate visual perception.

### Socioemotional selectivity theory

Carstensen et al. (1999) have proposed that older adults become more sensitive to positively valenced emotional stimuli because of social contexts and motivation. This model is termed Socioemotional Selectivity Theory (SST). These researchers postulated that this positive bias is due to the fact that seniors have less remaining life expectancy, so they tend to identify negative experiences as having less useful information, and, therefore, attribute more emphasis on positive affect (known as the “late positivity effect”). Some researchers have suggested that older adults become more efficient at behavioral regulation of physiological responses to emotional stimuli than do younger adults (Carstensen and Mikels, 2005; Mather and Carstensen, 2005). For example, Mather et al. (2004) reported that older adults had reduced fMRI amygdalar activation for negative pictures relative to younger adults (but not so for positive pictures). Also, LeClerc and Kensinger (2008) found greater fMRI activation in the VMPFC for positive emotional stimuli for older adults but for negative emotional stimuli for younger adults. The reason that SST is pertinent to the present study is because it predicts that older adults' positivity bias is the result of a later-life positivity bias accomplished through emotional regulation—rather than due to a neural deficit occurring earlier in the ventral affective system. The present study provides a mechanism to test the emotion perception deficit hypothesis of Allen et al. (2005) and the SST of Carstensen et al. (1999). This is because the two models make different predictions with regard to how P1 ERP amplitude will vary across younger and older adults using a PRP paradigm. The emotion perception deficit model predicts, in at least some older adults, that the ventral affective system declines and that these changes impair emotion perception and emotional decision making. Thus, this model predicts age differences in P1 amplitude for emotional facial discrimination. Specifically, younger adults should show larger P1 effects for angry faces than for happy faces (based on Holmes et al., 2008), but that older adults should show no emotional valence effects. In other words, it is predicted that younger adults will show emotional modulation of the P1 perceptual component, but that older adults will show just a perceptual response not modulated by emotional valence. Alternatively, the SST predicts that older adults experience a developmental change resulting in better emotional regulation so that negative emotions are inhibited allowing positive emotions to be more pronounced. Consequently, the SST predicts an attenuated effect for older adults for negative emotional faces but a stronger emotional response by older adults to positive emotional faces (e.g., Mather et al., 2004; LeClerc and Kensinger, 2008). Also, older adults should show higher P1 amplitudes for happy faces than for angry faces, whereas younger adults should show the opposite effect (LeClerc and Kensinger, 2008).

## The present study

**Figure 2:**
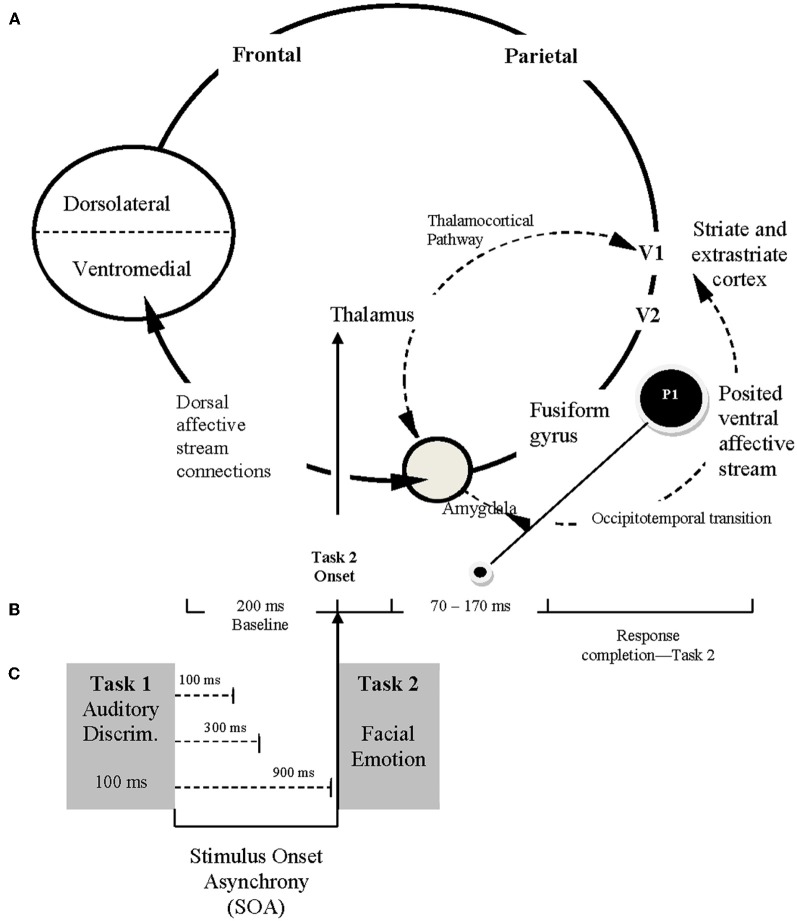
**Conceptual framework for ERP study. (A)** The posited neural framework involved in processing facial emotion. The ventral affective stream (posterior system) involves reciprocal and neuromodulatory influences between the amygdala and extrastriate-fusiform gyrus network (occipitotemporal transition). The dorsal affective stream (anterior system), includes the amygdala and higher order interactions with prefrontal cortical regions (less emphasized in the current study). **(B)** Schematic illustration of the time course for ERP recordings, reflecting the 200 ms baseline captured prior to Task 2 presentation and the 70–170 ms period immediately after Task 2 presentation during which P1 is recorded (O1–O2 electrodes). **(C)** The sequence of Task 1 and Task 2 presentation, emphasizing the relationship among Task 2 onset (following a specific stimulus onset asynchrony [SOA]), the temporal framework for ERP recordings, and the capture of P1 from occipital O1 and O2 electrodes.

The P1 ERP component is the peak associated with an early visual perceptual response that can be modulated by emotional valence. Our basic model is illustrated in Figure 2. This model (based upon Allen et al., 2005, 2011; Holmes et al., 2008, 2009a,b; Baena et al., 2010; Rotshtein et al., 2010; Dolcos et al., 2011) includes a “posterior portion” as well as an “anterior portion.” The posterior portion of the model includes the primary (V1) and secondary (V2) visual cortices, the fusiform gyrus (areas known to be closely associated with face perception), and the amygdala (the brain location most often associated with emotional arousal/activation, see Figure 2A). Conceptually, the anterior portion of the model includes the amygdala (the emotional “accelerator”), VMPFC (socioemotional control), and the dorsolateral prefrontal cortex (cognitive control). The present study is designed to examine the posterior portion of the model while largely attenuating (because of the 70–170 ms recording window of the P1) what is believed to be top-down feedback associated with the ventromedial and dorsolateral prefrontal cortex areas (i.e., the frontal portion) from biasing a more direct examination of amygdalar modulation of early visual perception (by using just an early ERP component).

The present ERP study with its emphasis on temporal precision can be used to replicate and extend the findings of fMRI study reported St. Jacques et al. (2010). Specifically, the present P1 ERP paradigm (refer to Figure 2B) allows a more temporally precise measure (the hemodynamic response in fMRI takes 2000 ms per stimulus to occur; Buckner, 1998) of their fMRI-based observation of an age deficit in functional connectivity between the amygdala and the bi-lateral visual cortex (our recording sites of O1 and O2 are located bi-laterally over the visual cortices). Our method also allows a distinction to be made between a perceptual deficit and what is posited to be an amygdalar modulation deficit. Evidence consistent with a perceptual deficit would be a main effect for age, and evidence consistent with an amygdalar modulation of visual perception would be an interaction involving age and emotion type (e.g., the logic used by Holmes et al., 2008, 2009a,b and Rotshtein et al., 2010). Note that we are assuming that early amygdalar activation is greater for negatively valanced emotional faces than positively valenced emotional faces, and this seems to be consistent with Holmes et al. (2008, 2009a,b)—who emphasized the importance of negatively valenced faces.

## Predictions

There are two categories of predictions in the present study, conceptually embellished in Figure 2. First, if emotion perception can occur automatically (without central attention), then either RT or accuracy for Task 2 (facial emotion discrimination task) should show progressively smaller task difficulty effects as SOA decreases (an SOA × Difficulty interaction, refer to Figures 1 and 2C), or the P1 effect should remain constant across SOA (or the shortest SOA should show at least as high of P1 amplitude as the longest) (Shaw et al., 2011). If increased adult age moderates attentional effects, then the aforementioned effects should interact with age.

The second category of predictions concerns whether SST (e.g., Carstensen et al., 1999) or the emotion perception deficit model (Allen et al., 2005; Denburg et al., 2005) better fit the present behavioral and/or electrophysiological (P1) results. As noted above, SST predicts better performance for older adults on happy faces than on angry faces, but the reverse for younger adults (Mather et al., 2004; LeClerc and Kensinger, 2008). On the other hand, the emotion perception deficit model predicts that older adults are especially likely to show a performance deficit for angry faces (relative to younger adults), but similar performance to younger adults on happy faces (Baena et al., 2010). That is, younger adults should show a larger P1 effect for angry faces than for happy faces, but older adults should show similar P1 effects for both angry and happy faces.

## Method

### Participants

There were 14 younger adults (10 female) and 14 older adults (7 female) who participated in this study. Data from one older adult were excluded due to low accuracy (<80%). Thus, a total of 14 younger and 13 older adults were included in the final data analyses. Younger adults were undergraduates at Oregon State University who participated in exchange for extra course credit. Their mean age was 19 years (range: 18–23 years). Older adults were individuals who resided in nearby communities. They were paid $20 for their participation. Their mean age was 70 years (range: 61–85 years). All participants reported having normal or corrected-to-normal visual acuity. None reported having any cognitive, neurophysiological dysfunction.

### Apparatus and stimuli

Stimuli were presented on an IBM-compatible microcomputer connected to a 19-in. ViewSonic monitor and were viewed from a distance of about 55 cm. The Task-1 stimulus was a pure tone or white noise (22 kHz, 8 bits, and 100 ms duration) and was presented via speakers on both sides of the computer monitor. The Task-2 stimuli contained one picture in the center of the screen, which subtended a visual angel of 6.23° (width) × 8.79° (height). There were 40 pictures with different actors (four categories: 10 male/angry, 10 male/happy, 10 female/angry, and 10 female/happy) taken from Tottenham et al. (2009). Within each category, the emotion expression was easy to determine for half of the faces (the easy condition) and was difficult to determine for the other half of the faces (the difficult condition; see Tomasik et al., 2009, for details). Each face was presented 30 times (excluding practice trials) per participant. For both tasks, manual responses were collected using a response box containing five buttons labeled 1–5 from left to right.

### Design and procedure

Each trial started with the presentation of the fixation display for 800 ms. The Task-1 auditory stimulus then sounded for 100 ms. After one of three SOAs (100, 300, or 900 ms) randomized within blocks, the Task-2 picture appeared in the center until the participant responded.

For Task 1, participants were asked to press the button labeled “1” with their left-middle finger for a pure tone and press the button labeled “2” with their left-index finger for a white noise (similar to a hissing sound). For Task 2, participants were instructed to respond to the emotion expression of the face. They were asked to press the button labeled “4” with their right-index finger for angry faces and press the button labeled “5” with their right-middle finger for happy faces. They were asked to respond to Task 1 and Task 2 quickly and accurately. Also, they were asked to respond to Task 1 before Task 2. Immediately after a response was recorded, the next trial began with the 800-ms fixation display.

Participants performed one practice block of 24 trials, followed by 15 experimental blocks of 80 trials each (a total of 1200 experimental trials). After each block, participants received a summary of their mean RT and accuracy for that block and were encouraged to take a break.

### EEG recording and analyses

The EEG activity was recorded using Q-cap AgCl electrodes from F3, Fz, F4, C3, Cz, C4, P3, Pz, P4, O1, Oz, O2, T7, T8, P7, P8, PO7, and PO8. These sites and the right mastoid were recorded in relation to a reference electrode at the left mastoid. The ERP waveforms were then re-referenced offline to the average of the left and right mastoids (see Luck, 2005). The horizontal electrooculogram (HEOG) was recorded bipolarly from electrodes at the outer canthi of both eyes, and the vertical electrooculogram (VEOG) was recorded from electrodes above and below the midpoint of the left eye. Electrode impedance was kept below 5 kΩ. EEG, HEOG, and VEOG were amplified using Synamps2 (Neuroscan) with a gain of 2,000 and a bandpass of 0.1–50 Hz. The amplified signals were digitized at 500 Hz.

Trials with possible ocular artifacts were identified in two steps (see also Lien et al., 2008). First, trials with ocular artifacts were rejected automatically using a threshold of ± 75 μV for a 1400 ms epoch beginning 200 ms before Task-2 stimulus onset to 1200 ms after Task-2 stimulus onset. Next, each of these candidate artifact trials was inspected manually. Rejection of trials with ocular artifacts in the EEG data led to the elimination of 5% of trials, but no more than 19% for any individual participant.

To quantify the overall magnitude of the P1 effect, we focused on the time window 70–170 ms after Task-2 stimulus onset. Specifically, the P1 effect was measured as the mean amplitude during this time window for electrode sites O1 and O2, relative to the mean amplitude during a 200-ms baseline period prior to Task-2 stimulus onset.

## Results

In addition to trials with ocular artifacts, we excluded trials from the final analyses of behavioral data (RT and proportion of errors; PE) and EEG data if RT for Task 1 (RT1) or Task 2 (RT2) was less than 100 ms or greater than 3000 ms (0.7% of trials for younger adults and 0.5% of trials for older adults). Trials were also excluded from RT and EEG analyses if either response was incorrect. Analysis of variance (ANOVA) was used for all statistical analyses (see below for details), with an alpha level of 0.05 to ascertain statistical significance. The *p*-values were adjusted using the Greenhouse-Geisser epsilon correction for nonsphericity, where appropriate.

### Behavioral data analyses

Data were analyzed as a function of age group (younger vs. older adults), Task 2 difficulty (easy: an extreme version of an emotional face vs. difficult: a morphed face that was in between a given emotional expression and neutral), Task 2 emotion (angry vs. happy), and SOA (100, 300, or 900 ms). Age group was a between-subject variable, whereas others were within-subject variables. Tables 1 and 2 show mean RT and PE for Task 1 and Task 2, respectively.

**Table 1:** **Mean response time (RT in ms) and proportion of errors (PE) for Task 1 as a function of age group (younger vs. older), Task-2 difficulty (easy vs. difficult), Task 2 emotion (angry vs. happy), and stimulus onset asynchrony (100, 300, and 900 ms)**.

	**Stimulus onset asynchrony**					
	**100 ms**		**300 ms**		**900 ms**	
	**RT**	**PE**	**RT**	**PE**	**RT**	**PE**
**YOUNGER**						
Easy						
Angry	655 (37)	0.072 (0.016)	624 (30)	0.038 (0.011)	643 (44)	0.028 (0.009)
Happy	655 (39)	0.053 (0.012)	639 (33)	0.030 (0.009)	638 (44)	0.027 (0.008)
Difficult						
Angry	652 (40)	0.059 (0.014)	629 (34)	0.039 (0.012)	652 (44)	0.025 (0.010)
Happy	647 (35)	0.053 (0.013)	631 (41)	0.039 (0.011)	636 (41)	0.026 (0.008)
**OLDER**						
Easy						
Angry	770 (47)	0.024 (0.008)	702 (54)	0.019 (0.005)	622 (33)	0.018 (0.008)
Happy	736 (40)	0.031 (0.007)	694 (46)	0.021 (0.005)	639 (42)	0.016 (0.004)
Difficult						
Angry	746 (44)	0.026 (0.007)	708 (45)	0.018 (0.007)	621 (34)	0.018 (0.008)
Happy	731 (45)	0.019 (0.006)	679 (44)	0.018 (0.006)	623 (36)	0.017 (0.005)

Note: The standard error of the mean is shown in parentheses.

**Table 2:** **Mean response time (RT in ms) and proportion of errors (PE) for Task 2 as a function of age group (younger vs. older), Task-2 difficulty (easy vs. difficult), Task 2 emotion (angry vs. happy), and stimulus onset asynchrony (100, 300, and 900 ms)**.

	**Stimulus onset asynchrony**					
	**100 ms**		**300 ms**		**900 ms**	
	**RT**	**PE**	**RT**	**PE**	**RT**	**PE**
**YOUNGER**						
Easy						
Angry	966 (47)	0.063 (0.020)	771 (36)	0.061 (0.027)	588 (23)	0.050 (0.015)
Happy	1016 (53)	0.110 (0.016)	828 (47)	0.099 (0.023)	623 (27)	0.092 (0.023)
Difficult						
Angry	1004 (51)	0.079 (0.022)	790 (42)	0.076 (0.020)	632 (28)	0.070 (0.017)
Happy	1039 (50)	0.149 (0.028)	843 (53)	0.121 (0.037)	665 (34)	0.124 (0.026)
**OLDER**						
Easy						
Angry	1249 (46)	0.023 (0.005)	998 (49)	0.032 (0.006)	731 (22)	0.029 (0.005)
Happy	1223 (34)	0.058 (0.016)	1006 (47)	0.039 (0.010)	28 (28)	0.039 (0.010)
Difficult						
Angry	1295 (38)	0.096 (0.025)	1082 (44)	0.092 (0.025)	818 (21)	0.115 (0.032)
Happy	1285 (44)	0.057 (0.016)	1051 (46)	0.056 (0.011)	775 (32)	0.037 (0.012)

Note: The standard error of the mean is shown in parentheses.

**Figure 3:**
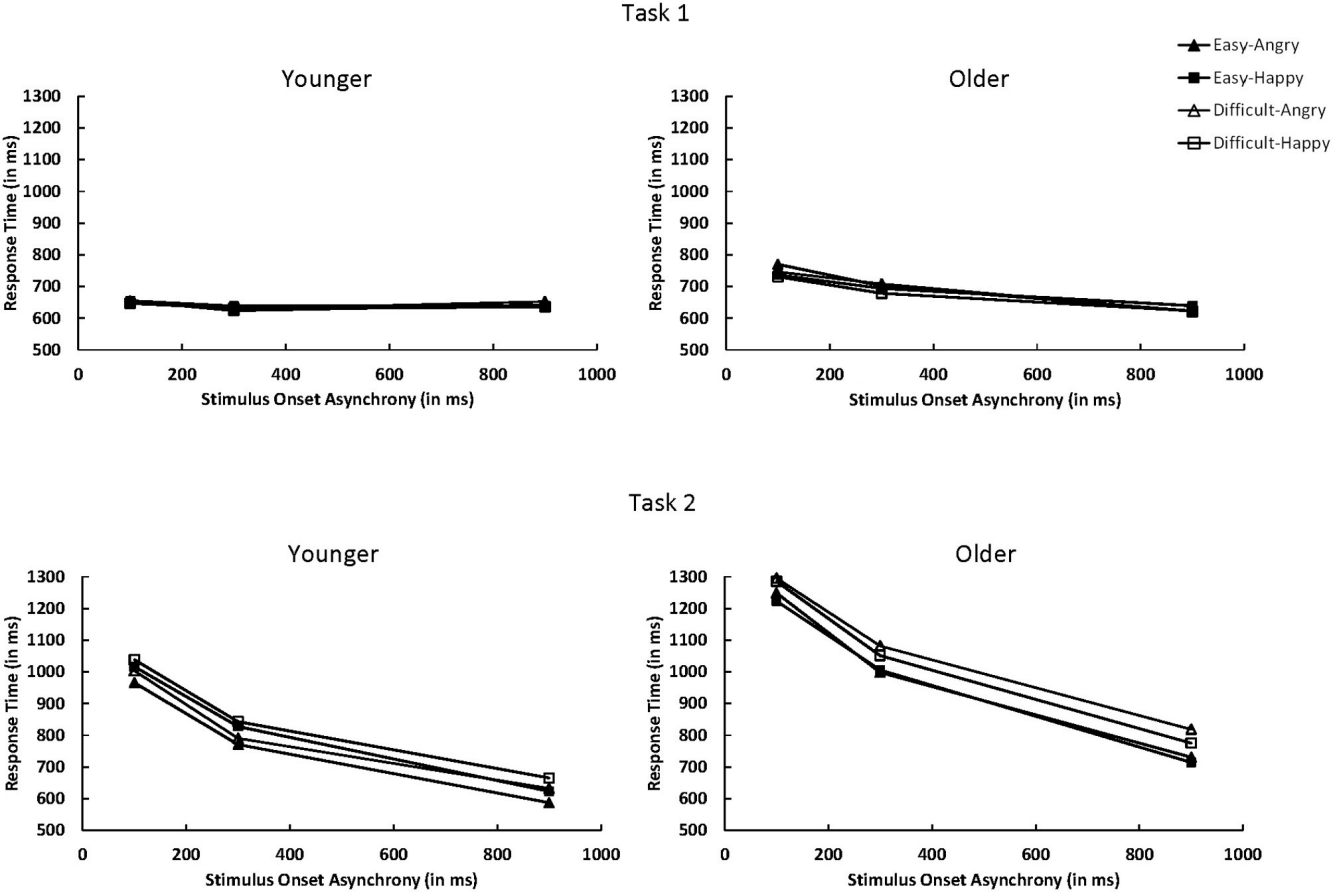
**Mean response time for Task 1 and Task 2 as a function of age group (younger vs. older), Task 2 difficulty (easy vs. difficult), Task 2 emotion (angry vs. happy), and SOA (100, 300, or 900 ms).** SOA, stimulus onset asynchrony.

For Task 1, RT1 decreased as SOA increased, *F*_(2, 50)_ = 8.46, *p* < 0.001, η^2^_*p*_ = 0.25 (see Figure 3). This decrease was more pronounced for older adults than younger adults, *F*_(2, 50)_ = 6.48, *p* < 0.01, η^2^_*p*_ = 0.21. Mean RT1 was 9 ms slower when Task 2 was an angry face (668 ms) than when it was a happy face (659 ms), *F*_(1, 25)_ = 4.47, *p* < 0.05, η^2^_*p*_ = 0.15. The three-way interaction between age, Task 2 emotion, and SOA was significant, *F*_(2, 50)_ = 3.80, *p* < 0.05, η^2^_*p*_ = 0.13. Older adults exhibited longer RT1 at short SOAs when Task 2 was an angry face than a happy face (difference in RT1 = 25, 18, and −10 ms at the 100, 300, and 900 ms SOAs, respectively), whereas no consistent pattern was observed for younger adults (difference in RT1 = 3, −8, and 11 ms at the 100, 300, and 900 ms SOAs, respectively).

Task 1 PE (PE1) decreased as SOA increased, *F*_(2, 50)_ = 24.10, *p* < 0.0001, η^2^_*p*_ = 0.49. In contrast to RT, this decrease was more pronounced for younger adults than older adults, *F*_(2, 50)_ = 9.04, *p* < 0.001, η^2^_*p*_ = 0.27. No other effects were significant.

For Task 2, the overall RT2 was longer for older adults (RT2 = 1019 ms) than younger adults (RT2 = 814 ms), *F*_(1, 25)_ = 15.08, *p* < 0.001, η^2^_*p*_ = 0.38. A large PRP effect of 439 ms on RT2 was observed, *F*_(2, 50)_ = 451.73, *p* < 0.0001, η^2^_*p*_ = 0.95. The PRP effect was larger for older adults (503 ms) than younger adults (379 ms), *F*_(2, 50)_ = 9.73, *p* < 0.001, η^2^_*p*_ = 0.28. RT2 was 47 ms longer in the difficult condition (940 ms) than in the easy condition (893 ms), *F*_(1, 25)_ = 100.46, *p* < 0.0001, η^2^_*p*_ = 0.80 (see Figure 3). The difficulty effect was larger for older adults (64 ms) than younger adults (30 ms), *F*_(1, 25)_ = 13.13, *p* < 0.01, η^2^_*p*_ = 0.34. Older adults had longer RT2 for angry faces than happy faces (1029 ms vs. 1009 ms), whereas younger adults had longer RT2 for happy faces than angry faces (836 ms vs. 792 ms), *F*_(1, 25)_ = 13.00, *p* < 0.01, η^2^_*p*_ = 0.34.

As in Tomasik et al. (2009) study, the interaction of Task 2 difficulty and SOA on RT2 was not significant, *F*_(2, 50)_ = 2.22, *p* = 0.1195, η^2^_*p*_ = 0.08; the difficulty effect was 42, 41, and 59 ms at 100, 300, and 900 ms SOAs, respectively. However, there was a trend toward underadditivity because the difficulty effect was reduced by 17 ms. The additivity between Task 2 difficulty and SOA was similar for younger and older adults, *F* < 1.0. For younger adults, the difficulty effect was 30, 17, and 44 ms at the 100, 300, and 900 ms SOAs, respectively. For older adults, the effect was 54, 65, and 74 ms at the 100, 300, and 900 ms SOAs, respectively.

Task 2 PE (PE2) was 0.032 higher for the difficult condition than for the easy condition, *F*_(1, 25)_ = 28.10, *p* < 0.0001, η^2^_*p*_ = 0.53. As in RT2, older adults had higher PE2 for angry faces (0.065) than happy faces (0.048), whereas younger adults showed an opposite pattern (0.116 for happy faces and 0.067 for angry faces), *F*_(1, 25)_ = 6.35, *p* < 0.05, η^2^_*p*_ = 0.20. The three-way interaction between age group, Task 2 difficulty, and Task 2 emotion was significant, *F*_(1, 25)_ = 6.07, *p* < 0.05, η^2^_*p*_ = 0.10. For older adults, higher PE2 for angry faces than happy faces was evident in the Task 2 difficult condition (0.101 vs. 0.050, respectively) but not in the easy condition (0.028 vs. 0.045). For younger adults, higher PE2 for happy faces than angry faces was evident in both the easy condition (0.100 vs. 0.058) and the difficult condition (0.131 vs. 0.075). No other effects were significant.

### ERP analyses

**Figure 4:**
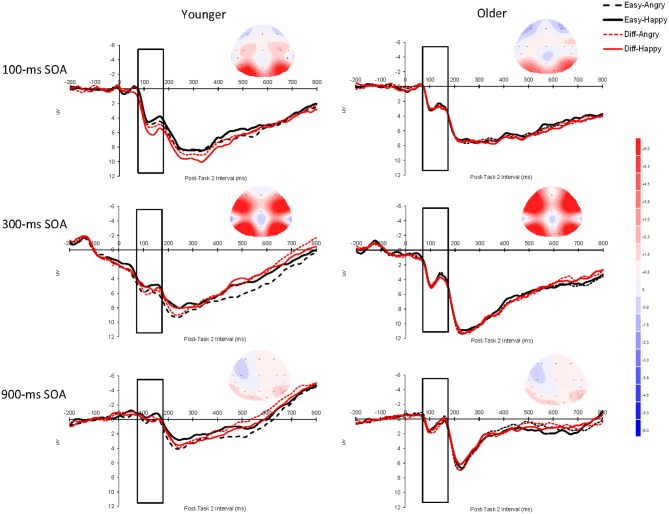
**Grand average event-related brain potentials (ERPs) for P1 elicited by Task 2 as a function of Task 2 difficulty (easy vs. difficult), Task 2 emotion (angry vs. happy), and SOA (100, 300, or 900 ms) for younger adults and older adults averaged across the electrodes O1 and O2.** The unfilled rectangular boxes indicate the time window used to assess P1 (70–170 ms after Task-2 stimulus onset). The scalp topography of the ERPs was obtained during the time window 70–170 ms after Task 2 stimulus onset for each SOA. The baseline period was the 200 ms prior to Task-2 stimulus onset. Negative is plotted upward and time zero represents Task-2 stimulus onset. SOA: stimulus onset asynchrony.

The P1 data analyses focused on the time window of 70–170 ms after Task-2 stimulus onset (Rotshtein et al., 2010, used 100–150 ms, but we slightly extended this to 70–170 ms, see Figure 2B). The P1 data were analyzed as a function of age group (younger vs. older adults), Task 2 difficulty (easy vs. difficult), Task 2 emotion (angry vs. happy), hemifield (left [O1 electrode] vs. right [O2 electrode]), and SOA (100, 300, or 900 ms). Figure 4 shows these P1 effects averaged across the electrodes O1 and O2. For each participant, there were a total of 1200 experimental trials. With the variables of SOA (3 levels), Task 2 Difficult (2 levels), and Task 2 Emotion (2 levels), there were a total of 100 observed trials for each SOA before trials that fell outside our RT cutoff or showed ocular artifacts were rejected.

The overall P1 effect was larger at the 300 ms SOA (5.357 μV) than at the 100 ms SOA (3.240 μV) or the 900 ms SOA (0.812 μV), *F*_(2, 50)_ = 54.15, *p* < 0.0001, η^2^_*p*_ = 0.68. This interpretation of the main effect was confirmed by post-hoc pairwise tests—300 ms vs. 100 ms, *F*_(1, 25)_ = 27.25, *p* < 0.0001; 300 ms vs. 900 ms, *F*_(1, 25)_ = 89.65, *p* < 0.0001; 100 ms vs. 900 ms, *F*_(1, 25)_ = 32.61, *p* < 0.0001. The interaction between Task 2 difficulty and Task 2 emotion was significant, *F*_(1, 25)_ = 4.48, *p* < 0.05, η^2^_*p*_ = 0.15; Angry faces elicited a larger P1 effect than happy faces in the easy condition (3.218 μV vs. 2.909 μV, respectively) but a similar P1 in the difficult condition (3.160 μV vs. 3.203 μV, respectively). This pattern was further qualified by a Group × Difficulty × Emotion interaction, *F*_(1, 25)_ = 6.65, *p* < 0.05, η^2^_*p*_ = 0.21 (younger adults: easy: angry = 3.67 μV, happy = 3.08 μV, difficult: angry = 3.45 μV, happy = 3.64 μV; older adults: easy: angry = 2.77 μV, happy = 2.74 μV, difficult: angry = 2.87 μV, happy = 2.77 μV).

To interpret this three-way interaction, we ran separate simple effects analyses by Task 2 difficulty. For easy trials, the Group × Emotion interaction was significant, *F*_(1, 25)_ = 4.67, *p* < 0.05, so younger adults did show a larger emotion effect than older adults. For difficult trials, though, the Group × Emotion interaction was not significant, *F*_(1, 25)_ = 2.10, *p* = 0.16. To clarify the Group × Emotion simple effect for easy trials, we ran separate analyses across age group. For younger adults, there was a simple effect of emotion, *F*_(1, 13)_ = 5.13, *p* < 0.05 (angry = 3.67 μV, happy = 3.08 μV), but the simple effect for emotion was not significant for older adults (*p* = 0.77) (angry = 2.77 μV, happy = 2.74 μV). This means that younger adults showed significantly higher amplitude P1 components for angry faces than for happy faces, but that there was no difference in emotional valance for older adults.

There was also a Difficulty × Hemifield interaction, *F*_(1, 25)_ = 10.16, *p* < 0.01, η^2^_*p*_ = 0.29. This interaction occurred because for the left hemifield (the electrode O1), difficult trials showed higher amplitudes (3.355 μV) than easy trials (3.092 μV), but for the right hemifield (the electrode O2), easy (3.284 μV) and difficult (3.308 μV) trials showed similar amplitudes. No other effects reached statistical significance. Finally, because our prediction was that older adults should be especially likely to show lower amplitudes at the 100 ms SOA than at longer SOAs, and we did observe a Group × SOA interaction that approached significance, *F*_(1, 25)_ = 2.37, *p* = 0.11, we analyzed the data separately by SOA. We observed simple effect of age that approached significance at the 100 ms SOA, *F*_(1, 25)_ = 3.47, *p* = 0.07 (younger = 4.20 μV, older = 2.82 μV), but not at the 300 ms SOA (*p* = 0.41) (younger = 5.88 μV, older = 4.84 μV) or the 900 ms SOA (*p* = 0.55) (younger = 0.67 μV, older = 1.07 μV).

## Discussion

The present study provided both ERP evidence for age-related sparing of attentional capacity (i.e., automatic processing) and for an age-related deficit in the processing of angry faces on trials with more pronounced emotional expressions (i.e., “easy” trials—in contrast to trials with faces with less pronounced emotional expressions, or “difficult” trials—these stimuli were closer to neutral). That is, younger adults showed significantly higher P1 amplitude for angry, easy trials than for happy easy trials, but older adults showed almost identical P1 amplitudes for both angry and happy easy trials. These ERPs results, then, replicate the results of St. Jacques et al. (2010) who used fMRI methods and observed a functional connection deficit for older adults (in the circuit connecting the top–down feedback loop from the amygdala to the early visual processing areas (primary and secondary cortices). On the other hand, we observed typical behavioral effects for a PRP task. We will first discuss the behavioral results and then the ERP data.

### Behavioral findings

For Task 2, both age groups showed PRP effects for RT2, although older adults showed larger PRP effects than did younger adults. PRP effects are thought to measure the delay in access to Task 2 response selection while individuals are completing processing on Task 1 response selection at short SOAs (Pashler, 1984). Thus, the larger PRP effect for older adults than younger adults reflects the central bottleneck to be larger for older adults than for younger adults, which is quite common (e.g., Allen et al., 2002, 2009; Lien et al., 2006). Another finding from the RT2 data was that older adults were faster in responding to happy than to angry faces, but younger adults were faster in responding to angry than to happy faces. This is a slight departure from earlier studies on single-task, facial emotional discrimination such as Baena et al. (2010) who observed that both age groups showed faster responses to happy faces than to angry faces, but that the effect was more exaggerated for older adults. Finally for Task 2, we observed additivity for RT2 between SOA and task difficulty, with a trend toward underadditivity—a result that replicated Tomasik et al. (2009). This is tempered behavioral evidence that processing stage before response selection for Task 2 emotions cannot be processed in parallel with response selection for Task 1 tones (because of the trend toward underadditivity). However, the trend toward underadditivity for RT along with similar P1 amplitudes at short and long SOAs do suggest that information for Task 1 and Task 2 can be processing simultaneously. Also, behavioral indices of performance may be controlled by later dorsal attentional stream processes, and it may not be possible to eliminate the structural bottleneck when both tasks must be processed with the dorsal attentional stream.

For RT1, there was a backward correspondence effect (Lien and Proctor, 2000). That is, RT1 was affected by Task 2 emotion type (RT1 was slower if Task 2 involved an angry face compared to a happy face). Backward correspondence effects are considered to be evidence of parallel processing (Lien and Proctor, 2000). The slight increase in RT1 as SOA decreased was offset by the reverse effect for Task 1 errors. Thus, there appeared to be no appreciable effect for SOA on Task 1.

### ERP findings

As noted in the Introduction, Shaw et al. (2011) found that the N2pc effect (a measure of spatial attention) was not modulated by SOA (and all amplitudes were significantly higher than zero). Also, these investigators found a stronger effect for angry faces than for happy faces (although this was a between-subjects effect). This was interpreted by Shaw et al. of evidence of automatic emotional processing. In the present study, we used the P1 component because of evidence of its association to amygdalar activation in epilepsy patients (Rotshtein et al., 2010). The PRP logic for whether central attention is required for Task 2 emotion perception, though, is the same for both Shaw et al. (N2pc) and the present study (P1). The present Task 2 still involved faces with different emotions and we used three different SOAs between Task 1 and Task 2, except the present study tested both younger and older adults (the Shaw et al. study presented two adjacent faces and asked participants to make a gender discrimination of a given emotion type, or the location discrimination of a given emotion type rather than directly making an emotional discrimination). In the present study, the amplitude for the 100 ms SOA was significantly higher than the amplitude from the 900 ms SOA. This finding suggests that participants were apparently able to process emotional faces at the 100 ms SOA. This leaves two unresolved issues, though. First, why was the amplitude for the 300 ms SOA higher than the amplitude for the 100 ms SOA? One possibility is that automaticity is graded (Pessoa et al., 2002). That is, perhaps at the 100 ms SOA individuals have enough amygdalar activation to carry out facial discrimination, but that at the 300 ms SOA there is an even stronger level of amygdalar activation. However, this graded interpretation is complicated by the fact that the P1 effect was significantly larger at the 100 ms SOA than the 900 ms SOA. Finally, and probably most perplexing, why was the P1 effect so small at the 900 ms SOA? An understanding of this drop in the P1 effect at 900 ms SOA relative to 100 ms and 300 ms SOAs will take additional empirical work to interpret, although we did observe clear evidence of Task 2 P1 effects at a short SOA (100 ms) that are consistent with the idea that individuals can process Task 2 stimuli simultaneously with the processing of Task 1 information for certain tasks (also see Shaw et al., 2011).

Another important issue to consider is why the behavioral data showed just a trend toward underadditivity (evidence for automatic emotion perception), but the ERP data showed stronger evidence consistent with emotion perception without central attention. As noted in Shaw et al. (2011), ERP components may be more sensitive than behavioral measures because they are a more direct measure of early emotional processing. We believe that this finding provides additional evidence of the efficacy of using ERPs to study attention and perception. These results are similar to those observed by Shafer et al. (2012). These investigators also observed evidence of both automatic emotional processing and non-automatic emotional processing in the same study (using fMRI methods).

It is important to note that the present P1 data cannot be easily accounted for by anything other than an emotional effect because emotion type interacted with age group and task difficulty. One might be concerned with the possibility that something like an early perceptual effect for Task 2 or even Task 1 modulation was driving the P1 effect. However, early perceptual effects for Task 2 were constant across different stimuli—the only thing that varied was emotion type. Also, Task 1 was a non-emotional task (pure vs. fuzzy tones). Thus, it is unclear how Task 1 could have modulated emotion type effects in Task 2.

### Theories of aging and emotion perception

As noted earlier, there are different theories of aging and emotion regulation. Clearly the most widely studied theory is the SST of Carstensen et al. (1999). This model proposes that older adults change their emotional regulation system to emphasize positive emotions and inhibit negative emotions. The present behavioral data seem to be partially consistent with this idea. Namely, younger adults processed angry faces faster than happy faces, but older adults showed the reverse effect. This is essentially the same pattern of results observed by Mather et al. (2004) and LeClerc and Kensinger (2008) using an fMRI paradigm. Thus, one possibility is that younger adults are maximally sensitive to the threat perception aspects of negatively valenced emotional stimuli (e.g., Allen et al., 2008), but that older adults are better able to block out negatively valenced stimuli (this would likely reflect an example of greater VMPFC executive control on the part of older adults). However, the electrophysiological ERP data from the present study showed a much more complicated picture of the processing dynamics of emotional facial discrimination. In particular, older adults showed no difference in P1 amplitude for happy and angry faces, but younger adults observed the more typical pattern of significantly larger P1 amplitude for angry faces than for happy faces on easy trials (e.g., see Holmes et al., 2008, 2009a,b). If older adults were better at emotional regulation such that positive emotional content was able to pass through the system more efficiently than negative emotional content, then one would predict higher-amplitude P1 effects for happy than for angry faces. One possibility is that the emotional regulation observed by Mather et al. (2004) and LeClerc and Kensinger (2008) occurs after the time period measured by the present P1 ERP component. Indeed, this view is consistent with Mather and Carstensen (2005) who claim an emotional regulation locus rather than an emotional arousal/activation locus of older adults' late positivity effect.

Another finding was that older adults, in general, showed a trend toward lower P1 amplitudes—especially at the 100 ms SOA (*p* < 0.07). These results seem to be more consistent with a more general drop in perceptual object activation in older adults (both in angry and happy faces). However, it is important to note that this trend toward a sensory deficit on the part of older adults cannot explain the emotional valance age difference observed for more pronounced emotional expressions (i.e., the Age Group × Difficulty × Emotion Type interaction).

### Are there age differences in amygdalar processing?

The present observation of emotional valence modulation of the P1 perceptual ERP effect for younger adults but not for older adults is consistent with the notion that younger adults exhibit amygdalar modulation of the visual cortices, but that older adults do not (see Rotshtein et al., 2010). This evidence is consistent with the fMRI functional connectivity results of St. Jacques et al. (2010) in which older adults appeared to exhibit a functional connectivity deficit in this circuit between the amygdala and the visual cortices. It is also consistent with the amygdalar volumetric loss for older adults that Fjell et al. (2009) observed using longitudinal methods. However, this view is not necessarily consistent with the meta-analysis results of Nashiro et al. (2012). Nachiro et al. reviewed the fMRI literature and failed to find evidence of an appreciable amygdalar decline with increased adult age. As illustrated in Figure 2, though, the amygdala is part of multiple systems involved in emotional processing. It can directly act on incoming stimulus information (the posterior portion of our model illustrated in Figure 2—emotional arousal/activation) as well as receiving top–down feedback from the prefrontal mechanism (the anterior portion of the model typically referred to as emotional regulation). Because the hemodynamic response used in fMRI takes 2 s to develop per stimulus (Buckner, 1998), fMRI research is likely measuring the amygdalar activation involving the top-down modulation by prefrontal mechanisms (the anterior portion of Figure 2) instead of the arousal of the amygdala by the visual cortices, and interactive modulation of the visual cortices by the amygdala that occurs in less than 200 ms (Cornwell et al., 2008; also see the anterior portion of Figure 2). Thus, it will take additional research on this topic to clarify the precise time course of potential age differences in amygdalar function. However, there is now DTI tractography evidence that the neural circuit connecting the amygdala with the fusiform gyrus and the visual cortices is the inferior longitudinal fasciculous (ILF) (Catani et al., 2003). Thus, this functional connectivity age difference observed by St. Jacques et al. (2010) and the differential modulation of emotional valence on visual perception across age observed on the P1 data in the present study could be a white-matter integrity deficit in the ILF neural pathway more than a deficit in amygdalar function, *per se*.

### Graded capacity sharing and recruitment

Another possible explanation of the present results is that Task 1 and Task 2 share processing resources (a graded capacity-sharing model). This sort of model would predict that the brain would recruit additional resources at shorter SOAs (when central attention would need to process both Task 1 and Task 2) relative to the longest SOA (900 ms—when Task 1 response selection would be completed before Task 2 was presented). Older adults tend to show more neural recruitment (as measured by PET scanning) than younger adults on some tasks (Grady et al., 1995, 1996; Cabeza et al., 1997; Madden et al., 1999), and this has been taken as evidence that older adults attempt to compensate for less efficient processing by recruiting more neurons. A recruitment model would seem to predict higher amplitudes at the 100 ms SOA than the 300 ms or 900 ms SOAs, and that older adults should show higher amplitudes than younger adults. While this interpretation is theoretically intriguing (because of the aging research on neural recruitment), the present P1 amplitudes for the 300 ms SOA were significantly higher than for the 100 ms SOA. Also, older adults did not exhibit higher P1 amplitudes than younger adults. However, without this disconfirming evidence, one might have claimed that perhaps the P1 component was really showing the attentional capacity allocated to Task 2 rather than to an emotional response (but see Holmes et al., 2008, 2009a,b). Overall, though, it does not appear that the present results are consistent with the attentional capacity (rather than emotional activation) interpretation.

### General sensory visual deficits

One potential interpretation of the present results is that older adults simply experienced a general sensory deficit. Indeed, Lindenberger and Baltes (1994) and Baltes and Lindenberger (1997) proposed a “common cause” model of aging in which a general/common sensory deficit mediated all age differences in cognition. However, other studies have provided both cross-sectional (e.g., Allen et al., 2001) and longitudinal (e.g., Anstey et al., 2001) evidence that there is unique age-related variance that cannot be accounted for by a common factor. Even though a precise test of this issue requires mediational analyses rather than the moderation analyzes afforded by ANOVA, the present Age Group × Difficulty × Emotion Type interaction does not appear to be consistent with a common-cause interpretation. First, a general sensory decrement in the visual cortices would have no mechanism to respond to emotional valance (the Nim Stim faces are equated on perceptual difficulty). This would seemingly require that the amygdala modulate visual cortex activation levels differentially across emotion type (e.g., Holmes et al., 2008, 2009a,b; Rotshtein et al., 2010). In particular, the finding that younger adults responded differentially to angry and happy less-distorted faces, but that older adults showed similar activation levels for both emotion types suggests that amygdalar modulation of the visual cortex occurred in the present study.

### Limitations and future research

The main limitation of the present research is a lack of generalization and replication. For example, does one observe this age deficit for negatively valenced stimuli that are faces, or would it generalize to other familiar perceptual objects (e.g., snakes vs. a mother holding her baby compared to angry vs. happy faces)? Future research that extends the P1 effect to other types of stimuli is needed. The work of Holmes et al. (2008, 2009a,b) does suggest that a similar pattern of results is obtained when one compares non-anxious and anxious participants (instead of younger and older adults), but it will still be important to replicate and extend the present age effects.

### Conflict of interest statement

The authors declare that the research was conducted in the absence of any commercial or financial relationships that could be construed as a potential conflict of interest.
